# 

**Published:** 1916-09-30

**Authors:** 


### PASSING EVENTS AND TOPICS.

### Sufficient Military Hospital Accommodation.

According to a letter received by the Keighley Guardians from the Army authorities, a sufficiency of hospital accommodation for the wounded has now been secured. The communication stated that in these circumstances there was no necessity to accept the offer of the Guardians to set apart a block at the infirmary for military cases.

### Chelsea Hospital for Women.

The Council of the Chelsea Hospital for Women are appealing for funds to complete their reconstruction scheme, to enable them to build a new nurses' home and thus provide accommodation in the hospital for eighty patients. Contributions should be sent to Mr. S. H. Goldsmid, hon. treasurer, or Mr. Herbert H. Jennings, Secretary, at the hospital as above.

### Music for the Sick and Wounded.

The Joint War Committee of the British Red Cross Society and of the Order of St. John have sent out to Malta one of Miss Lena Ashwell's concert parties, which are organised in conjunction with the Ladies' Auxiliary Committee of the Y.M.C.A. The party consists of six artistes, who will stay two months, and will give entertainments at the hospitals and convalescent camps on the island.

### A Zeppelin Bed.

The incredulity of a special constable on the night of the Zeppelin raid of September 2 appears to have been a valuable source of income to a certain hospital, for it is stated that so convinced was he that the story of the wrecked Zepp. was untrue that he backed his conviction with a £5 bet which, when afterwards paid over, was added to by his fellow-"specials" to such an extent that enough money was realised to endow a bed in the local institution.

### A "Barrett Roue Scholarship."

Dr. William Barrett Roué, recently deceased, has left £300 to the Bristol General Hospital for the purpose of founding a scholarship for medical students of the hospital, the scholarship to be called the "Barrett Roué Scholarship." Dr. Roué was himself an old and distinguished student at the hospital, in which he always took a warm interest. He practised for many years in Clifton, and was also well known as one of the physicians to the Bristol Royal Hospital for Sick Children and Women.

### Wounded as Pierrots.

Wounded soldiers from the Bristol Red Cross Hospital, Denbigh, recently gave what is described as a magnificent open-air performance to the hospital staff and public of the surrounding district. The first part of the programme was a pierrot performance, which was very well appreciated, all the popular songs and choruses being sung. The second half was a most amusing sporting sketch entitled "Dandy Dick," written by one of the patients. Each character was most cleverly represented, the whole company being actors of considerable merit, who have formed themselves into a company under the title of "The Jolly Boys." After the performance one of the patients thanked the audience, and said it had given them all great pleasure to prepare and perform their little sketch and thereby to return thanks for the kindness they had received from the hospital staff and residents of the district.

### HOSPITAL AND INSTITUTIONAL RESULTS IN 1915.

**Mary Yolland Home.**—This is an intermediate cottage home in connection with the Hospital and Home for Incurable Children at Northcourt, Hampstead, and was opened in 1910. Although but a small institution (eleven beds), the accounts are kept under the Uniform System. Income, £454; expenditure, £495.

**Western Counties Institution, Starcross** (for mental defectives).—There were 334 inmates at the close of the year—231 boys and 103 girls. Many instances of special improvement are mentioned in the superintendent's report. The institution is very little dependent upon voluntary support.

**Children's Country Holiday Fund.**—Only about half the normal number (20,000) of children were sent away in 1915, as the committee had forebodings as to the financial position. There was, however, a surplus of £3,119 on the year's working.

**Catherine Gladstone Free Convalescent Home, Mitcham.**—Three hundred and seventy men and boys and 506 women and girls were admitted. The home has been of assistance to a number of the Metropolitan hospitals. Total income £5,806 (including a legacy of £4,402); total expenditure, £2,169.

**King's College Hospital, Denmark Hill.**—The average number of beds in daily use was 653, of which 514 were occupied by military and 139 by civilian patients; the accommodation for the last-named class was increased towards the end of the year. The total attendances in the out-patient and casualty departments were 89,395. The expenditure exceeded the income by £8,224. As the deficit on the previous year's accounts was £12,361, the financial outlook is not promising, and a good response to the special appeal now being issued is earnestly hoped for by the committee. Subscriptions and donations have fallen off substantially. A large part of the hospital, together with some extra accommodation, forms the Fourth London Territorial Force General Hospital, and for this service £47,131 was paid by the War Office in 1915.

**Queen's Hospital for Children, Hackney Road.** The report is printed on poor paper, and the general "get-up" of the report is indifferent. This questionable economy is more open to objection than the exclusion of seventeen pages of unessential matter. In-patients increased from 1,736 to 2,029, out-patients from 41,892 to 42,225. The expenditure exceeded the income by £2,012.

**Sheffield Institution for the Blind.**—The institution has, by arrangement with the local Guardians, taken a number of itinerant musicians off the streets and is teaching them useful trades. On the school accounts the balance of £539 in hand in January 1915 was reduced to £62 by the end of the year; on the other hand the manufacturing account shows receipts amounting to £7,117, and an expenditure of only £6,530.

### Gifts to Hospitals.

The Royal Westminster Ophthalmic Hospital has received a grant of £400 from the trustees of the Zunz Bequest.

Under the will of Mr. Henry Howorth, a Rochdale printer, Rochdale Infirmary will benefit to the extent of about £4,000.

The Sunderland Royal Infirmary has received a donation of £100 from Mr. P. Lodwidge, a member of the House Committee.

Miss Helen Smith, of Chester, left £200 each to the National Hospital for the Paralysed and Epileptic and the Cancer Hospital, Brompton.

Mrs. Mary Sparrow, of Beckminster, Staffs, who died on June 20, left £500 each to the Wolverhampton Women's Hospital and the Wolverhampton and South Staffs Hospital.

Major H. E. Baskerville Walton, who died on May 22, left £250 to the Sussex County Hospital; he also left £500 to the British Ophthalmic Hospital at Jerusalem.

Major R. W. Richardson, of York, who died on April 22, has bequeathed £1,000 each to the York County Hospital and the York Dispensary; and £500 to the York Nurses' Home.

The late Canon Binney, of Chester, formerly vicar of Northwich, left £200 to Northwich Infirmary.

Mr. John Butters, of Derby, who died on January 6 last, left £100 each to the Derbyshire Royal Infirmary, the Derby Hospital for Women, and the Midland Deaf and Dumb Institute.

The late Mrs. Amy Yates, of Rhyl, left £250 to the Royal Alexandra Hospital, Rhyl, and £250 to the Red Cross Society.

Mr. Frederick Andrew, a Lincoln solicitor, who died on May 16 last, left £1,000 to the Lincoln County Hospital, and £500 to the Lincoln General Dispensary.

Mr. J. Fenwick Harrison, the Liverpool shipowner, has sent a donation of £1,000 to St. Bartholomew's Hospital.

For the endowment of three beds at the Princess Alice Memorial Hospital, Eastbourne, £3,000 has been left by Miss Sarah Diplock, of South Down Hall, Polegate, Sussex.

The Stockport Infirmary has received a legacy of £500 bequeathed by Mr. James Bardsley, of Hazel Grove, and a donation of £100 from Mr. James B. Hodgkinson, of Poynton Towers.

Rates of Subscription: Twelve months, 6/6; Six months, 4/-; Three months, 2/-, post free to any address in the United Kingdom. Abroad, Twelve months, 8/8; Six months, 5/-; Three months, 2/6, post free. The Hospital "Index" to Volume LIX., October 1915 to March 1916, is now ready, price 1½d. per copy, post free. Address The Manager, "The Hospital," The Hospital Building, 28/29 Southampton Street, Strand, London, W.C.

